# A comprehensive review and case series of adjuvant and neoadjuvant therapies in acral melanoma with emerging insights from CT DNA testing

**DOI:** 10.3389/fonc.2025.1619248

**Published:** 2025-10-14

**Authors:** Adityanarayan Rao, Akash Mathavan, Akshay Mathavan, Bentley Doonan

**Affiliations:** ^1^ Internal Medicine Department, University of Florida Division of Internal Medicine, Gainesville, FL, United States; ^2^ The Mayo Clinic, Jacksonville, FL, United States

**Keywords:** acral melanoma, neoadjuvant immunotherapies, adjuvant immunotherapy, CT DNA monitoring, melanoma

## Abstract

**Introduction:**

Acral melanoma is a rare but aggressive type of skin cancer that appears on the hands, feet, and under the nails. Unlike other melanomas, it is not linked to sun exposure and has unique genetic features that may require different treatment strategies. This research aims to explore whether immunotherapy given before surgery (neoadjuvant therapy) or after surgery (adjuvant therapy) is more effective in improving patient outcomes.

**Methods:**

By analyzing existing studies, we hope to understand which approach better reduces the risk of cancer returning and improves survival.

**Results:**

Further, we assessed the clinical course and outcomes of adjuvant and neoadjuvant immunotherapy through a case series of five patients who underwent either intervention. Additionally, we examine how new blood tests that detect tumor DNA could help track treatment response and personalize therapy.

**Discussion:**

Our findings may guide doctors in selecting the best treatment plans, leading to improved care for patients with this challenging disease and advancing research into more effective therapies.

## Introduction

Acral melanoma is a rare subtype of melanoma that primarily affects the palms, soles, and subungual areas. Unlike other forms of melanoma, acral melanoma is not strongly associated with ultraviolet radiation exposure. The unique characteristics and clinical behavior of acral melanoma necessitate distinct therapeutic approaches, including the potential roles of adjuvant and neoadjuvant immunotherapy.

Acral melanoma accounts for approximately 2%–3% of all melanoma cases in Caucasians but represents a higher percentage in darker skinned individuals, including those of African, Asian, and Hispanic descent (1). It shows an age adjusted incidence of 2.0 per million persons in the United States and the proportion of acral melanoma among all types of melanoma is highest in Black Americans where it constitutes 32.6% of all melanoma cases (2). A study by Curtin et al. (2005) showed that acral melanomas often have distinct genetic alterations compared to other melanoma subtypes, including a higher prevalence of KIT mutations (15%–20% of cases) and a lower frequency of BRAF mutations (less than 5%) (1). This suggests different etiological factors and potential therapeutic targets. In addition, a study by Curtin et al. (2006) found that mutations in the PI3K-AKT pathway were present in about 36% of acral melanomas, further emphasizing the unique molecular landscape of this melanoma subtype (3). These mutations have created possible targets for therapy such as Tyrosine kinase inhibitors that have potential in KIT-mutated melanomas (4).

This literature review aims to provide a comprehensive overview of acral melanoma and to examine the effectiveness of adjuvant versus neoadjuvant chemotherapy in its treatment. It further serves to highlight the dearth of literature regarding the treatment of acral melanoma and the need for further research.

## Clinical presentation and diagnosis

Acral melanoma often presents as a pigmented lesion on the palms, soles, or beneath the nails. Due to its location, it is frequently diagnosed at a more advanced stage compared to other melanomas. Phan et al. (2006) reported that approximately 50% of acral melanomas are diagnosed at Stage III or IV, highlighting the challenges in early detection which in turn lead to poor outcomes and a higher likelihood of metastasis and recurrence after treatment (5). Its atypical presentation leads to frequent misdiagnosis. And it can often mimic benign conditions like warts, calluses, or even fungal infections. Leading to diagnostic delays.

Dermoscopic features of acral melanoma include parallel ridge patterns and irregular pigmentation, which can aid in early diagnosis. Additional dermoscopic features include irregular blotches, asymmetry of structure and color as well as the absence of a furrow pattern. The BRAAFF pattern is often used in this process to assist in diagnostic accuracy. A meta-analysis by Williams et al. demonstrated that dermoscopy improved diagnostic accuracy and can lead to early detection (6). Early diagnosis is essential since acral melanoma is associated with the poorer prognosis at baseline even compared to other melanomas. Delay in diagnosis often results in a more advanced stage, increased tumor thickness and lower survival rates (7).

Diagnosis is confirmed through biopsy and histopathological examination, which typically reveals atypical melanocytes and a high mitotic rate, indicative of aggressive tumor behavior (8). Pathologically earlier diagnosis can be more challenging due to its atypical cellular features which might resemble benign proliferations.

## Treatment modalities

### Surgical management

The primary treatment for localized acral melanoma is surgical excision with appropriate margins. Walker et al. (2020) found that wider surgical margins and partial digit amputations are associated with no changes in overall survival or recurrence free survival compared to complete digit amputation allowing for less morbidity and sparing treatment (8). Sentinel lymph node biopsy (SLNB) is often performed to assess regional lymph node involvement, which is a critical factor in staging and prognosis. Their review indicated that SLNB positivity in acral melanoma can be as high as 40%, necessitating thorough staging and follow-up (8).

### Adjuvant therapy

Adjuvant therapy refers to additional treatment given after the primary surgery to reduce the risk of melanoma recurrence. The use of adjuvant therapy in acral melanoma, including immune checkpoint inhibitors (such as nivolumab and pembrolizumab) and targeted therapies (like imatinib for KIT-mutant melanomas), has shown promise in improving survival outcomes. Carvajal et al. (2011) showed the among patients with advanced melanoma (including acral, mucosal and chronically sun-damaged sites) with KIT mutations, a subset of patients showed clinical response, although this data was seen specifically in unresectable melanomas (9). Weber et al. (2017) demonstrated that adjuvant nivolumab improved recurrence-free survival (RFS) by 18% at 18 months compared to ipilimumab (10). Similarly, Eggermont et al. (2018) showed that adjuvant pembrolizumab improved 1-year RFS rates by 15% compared to placebo in patients with resected stage III melanoma (11). However, the role of adjuvant chemotherapy specifically in acral melanoma remains less defined. Even the above studies do not specify their data regarding acral melanoma and this lack of data represents a gap in the current evidence base. It emphasizes the need for further subgroup analysis or dedicated studies that focus exclusively on acral melanoma. Many studies suggest that traditional chemotherapeutic agents have limited efficacy in melanoma, leading to a preference for immunotherapy and targeted therapy in the adjuvant setting in current guidelines (12).

### Neoadjuvant therapy

Neoadjuvant therapy, administered before the primary surgical intervention, aims to reduce tumor size and eliminate micrometastases. In acral melanoma, neoadjuvant approaches, including chemotherapy, have been explored to improve surgical outcomes and overall survival. A study by Amaria et al. (2018) found that neoadjuvant immune checkpoint blockade resulted in a major pathologic response in 30%–50% of patients, which correlated with improved overall survival (12). Recent trials have investigated the efficacy of neoadjuvant immune checkpoint inhibitors and targeted therapies, demonstrating significant tumor regression and improved resectability (13). For instance, the study by Blank et al. (2018) showed that neoadjuvant ipilimumab plus nivolumab led to a higher rate of pathologic complete response (pCR) compared to the adjuvant setting (35% *vs*. 8%) (13). However, the role of traditional neoadjuvant immunotherapy remains controversial, with mixed results regarding its impact on long-term survival (14, 15). As discussed above with adjuvant therapy there is a dearth of literature regarding neoadjuvant therapy specifically for acral melanoma and as a result further study will be needed. Both Amaria et al. and Blank et al. primarily analyzed general cutaneous melanomas and data has been extrapolated for the purposes of treating acral melanomas in the absence of definitive studies.

## Methodology

**Figure 1:**
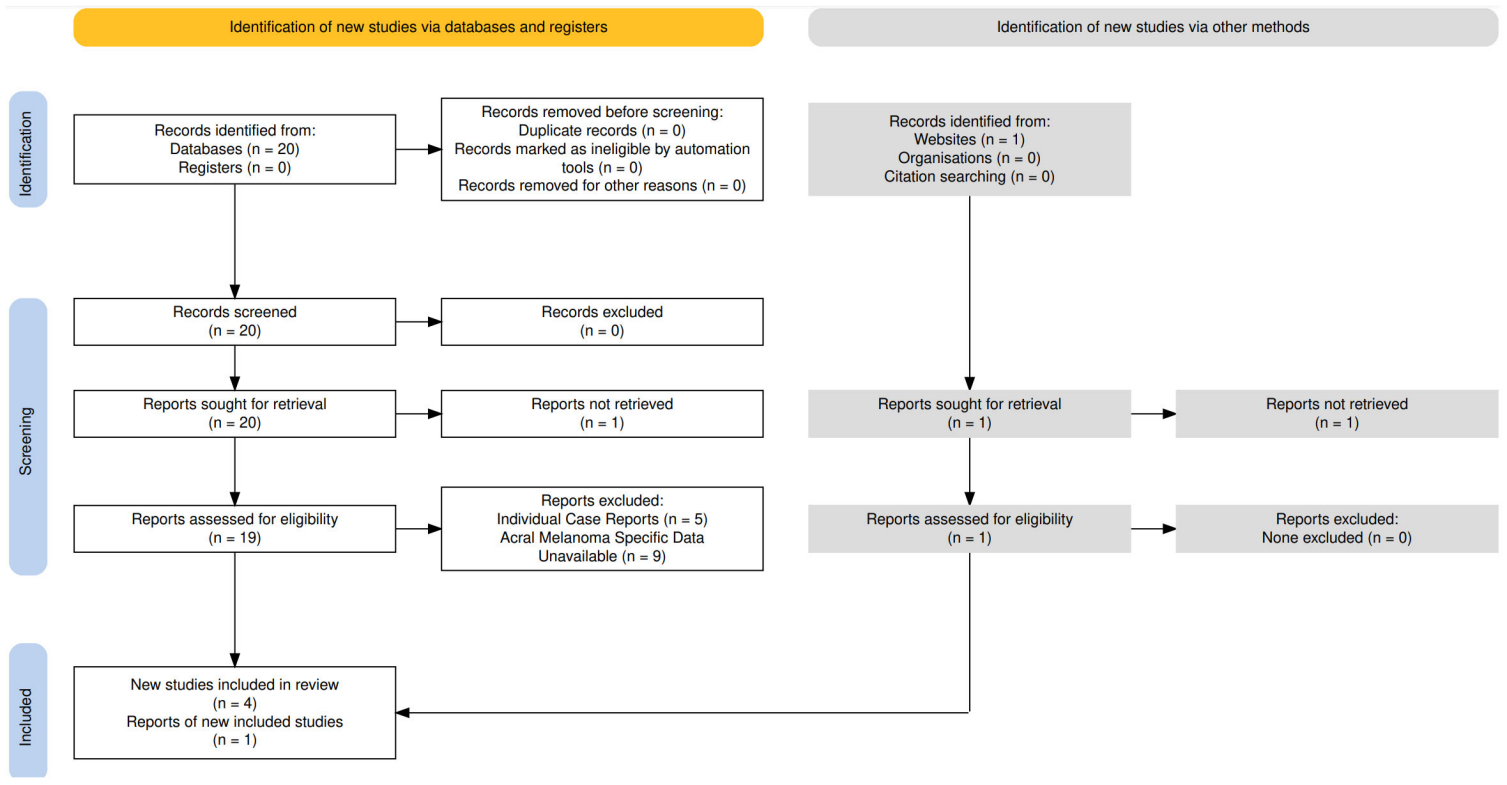
PRISMA diagram depicting the literature review of articles available in PubMed Database depicting the results of neoadjuvant and adjuvant immunotherapy in acral melanoma.

For this systematic review we searched PubMed Database from inception for randomized control trials, retrospective and prospective cohort studies that assessed the outcomes of acral melanoma patients using the key words “acral melanoma,” “adjuvant therapy,” “neoadjuvant therapy,” and “immunotherapy.” Studies were then screened to exclude any individual case reports or studies that did not include data on acral melanoma patients. Key endpoints in the evaluated studies included RFS, pathological complete response (pCR) and tumor downstaging. This process is depicted in the below PRISMA diagram (**Figure 1**).

## Results

The literature review returned 20 articles that were identified to contain the keywords discussed above. One was found to be a non-peer reviewed article that could not be retrieved for further evaluation. Of the remaining 19 articles, 5 were identified to be case reports with only a single patient focus and 9 were identified to have no available data regarding acral melanoma specifically and as such were excluded from further evaluation.

### Neoadjuvant studies

Neoadjuvant immunotherapy was evaluated in the SWOG Pembrolizumab Trial, which compared neoadjuvant plus adjuvant pembrolizumab to adjuvant-only pembrolizumab. The neoadjuvant-adjuvant approach significantly improved event-free survival (EFS) compared to the adjuvant-only group (*P* = 0.004), with 2-year EFS rates of 72% versus 49%, respectively (16). Treatment-related grade 3 or higher adverse events were similar between groups (12% *vs*. 14%) (16). However, among the nine patients with acral melanoma included in the study, no definitive conclusions could be drawn regarding the benefit of neoadjuvant pembrolizumab due to small sample size, though both deaths occurred in the adjuvant-only group (16).

### Adjuvant studies

Adjuvant immunotherapy studies in acral melanoma have produced mixed results. The SWOG Pembrolizumab Trial found no definitive benefit of neoadjuvant pembrolizumab in acral melanoma due to small sample sizes, but overall, adjuvant pembrolizumab improved event-free survival (16).

Li et al. performed a retrospective cohort analysis compared adjuvant PD-1 inhibitors with high-dose interferon α-2b (HDI) in Chinese patients with cutaneous and acral melanoma. For patients with acral melanoma, the PD-1 inhibitor group had a median relapse-free survival (RFS) of 7.0 months compared to 15.3 months in the HDI group (HR, 1.204; 95% CI [0.521–2.781]; *p* = 0.633) (17). No statistically significant differences were found in 6-month, 12-month, or 18-month RFS rates between the PD-1 inhibitor and HDI groups (17). The incidence of distant metastasis as the first recurrence at 18 months was 50% in the PD-1 inhibitor group and 29.2% in the HDI group (17).

Zhong et al. performed a retrospective study evaluated the efficacy of different adjuvant treatments for stage III BRAF V600 mutant melanoma in a Chinese population, though it did not provide specific results for acral melanoma (although they were included in the study data set). The study included 93 patients, with 25 receiving anti-PD-1 immunotherapy, 25 receiving dabrafenib plus trametinib (D+T), 23 receiving vemurafenib, and 20 under observation. Median RFS was not reached in the D+T group, 15 months in the vemurafenib and PD-1 groups, and 10 months in the observation group (18). D+T showed a statistically significant benefit in RFS compared to observation (*p* = 0.002) and better relapse control compared to anti-PD-1 monotherapy (*p* = 0.032) (18).

Arak et al. performed a retrospective analysis of 114 patients with stages III–IV acral melanoma, with a mean follow-up period of 40 months, and found that 56.1% received systemic adjuvant treatment (consisting of anti-PD-1 therapy, interferon, temozolomide and protein kinase inhibitors), with 48.4% receiving anti-PD-1 therapy (19). Disease-free survival (DFS) for patients receiving adjuvant therapy was 24.0 months, compared to 15.0 months for those who did not receive any adjuvant therapy (*P* = 0.051) (19). Median DFS could not be determined for patients receiving anti-PD-1 treatment due to a lack of events. Median DFS in patients who received temozolomide, interferon, and BRAF-MEK inhibitors were 23, 22, and 23 months respectively. Median overall survival (OS) was 71.0 months for patients receiving adjuvant therapy and 38.0 months for those who did not (*P* = 0.051) (19). No significant differences in OS were observed among different adjuvant treatments (38.0 months in those who received anti-PD-1 treatment compared to 33.0 months in those with other systemic adjuvant therapy (*P* = 0.765).

Mao et al. performed a randomized phase II trial compared 1 month versus 1 year of adjuvant high-dose interferon α-2b (HD-IFN) in high-risk (stages IIb–IIIc) acral melanoma patients. A total of 158 patients were enrolled, with 147 eligible for survival analysis. Median follow-up was 36.1 months, with median RFS of 17.9 months for the 1-month HD-IFN group (arm A) and 22.5 months for the 1-year HD-IFN group (arm B) (*P* = 0.72) (20). Stratified analysis showed statistically significant differences in RFS for patients in stage IIIb–IIIc between the two arms (*P* = 0.02) (20). Patients in arm B had higher incidences of reversible Grade 3/4 hepatotoxicity compared to arm A (*P* = 0.03) (20).

## Case series

Below, we discuss five cases of patients, three of which were managed with conservative therapy using gold standard imaging with an add on of CT DNA monitoring for enhanced surveillance while two others were managed in the neoadjuvant immunotherapy with the hope of down-staging in addition to CT DNA monitoring.

CT DNA monitoring was implemented via commercially available Signatera testing which uses multiplex PCR Next Gen sequencing created to track tumor specific somatic variants present in the patient’s plasma. Processing involves sequencing both patient’s tumor and their matched normal DNA that are then compared to identify the tumor specific variants (21). Blood samples are collected, centrifuged via a two-step protocol to separate plasma at a lower speed and higher speed to remove residual cellular debris and plasma is analyzed to detect these variants. Detection is reported quantitatively in mean tumor molecules per ml of plasma (MTM/ml) (21). Signatera testing was utilized due to commercial availability however not performed in a clinical trial but as an add-on to standard of care.

### CT DNA enhanced surveillance case 1

#### Clinical presentation

Patient A is a 76-year-old male who presented for the evaluation and management of concern for acral melanoma on the sole of his left foot. The patient originally noticed the bump while purchasing new shoes and was concerned for a plantar wart. He presented to dermatology who biopsied the lesion and found it to be acral melanoma.

#### Diagnostic workup

Surgical pathology confirmed amelanotic acral lentiginous malignant melanoma however depth of invasion could not be identified. He underwent a PET scan and an MRI that confirmed the localized nature of his disease. He was referred to surgical oncology and underwent WLE and SLE which showed negative margins, and a T3a lesion but NM lymphatic scan was not completed due to radiotracer penetration in an area too deep for safe resection. Final staging was identified as IIA pT3aNxMx.

#### Treatment strategy

After discussion with the patient regarding the lack of indication for immunotherapy treatment options in stage IIA and surveillance methodologies we opted for combined traditional surveillance with dermatology assessments, lower extremity imaging guided by orthopedic oncology as well as follow-up imaging as needed based on symptomology and adding on CT DNA enhanced monitoring every three months to the existing standard of care. Initial CT DNA was undetectable.

#### Outcome & follow up

Seven months after initial surgical management, patient’s ctDNA turned positive at a level of 0.08, and a PET scan was performed which showed a positive deep left psoas node indicative of regional spread. On discussion of management options including initiation of immunotherapy, surgical reresection, or high-dose radiation therapy in form of stereotactic body irradiation were presented. If definitive local therapy with surgery or radiation were to fail then systemic dual IO immunotherapy would be offered. Patient elected for SBRT to the lesion due to personal reasons and wanting to delay onset of immunotherapy, ctDNA uptrended to 0.14 MTM/ml at time of initiation of SBRT. Following treatment, the ctDNA down trended to 0.13 MTM/ml the following month and then down trended to undetectable two months later. Imaging at the time showed no evidence of disease. Patient continued on surveillance and subsequent monthly monitoring levels alternated between undetectable and borderline detectable disease (0.00, 0.19, 0.00, 0.08, 0.27, 0.09). Repeat imaging during this time period failed to show definitive evidence of recurrence and patient remained asymptomatic and elected for continued trending and observation. One year after the initial presentation patient is doing well and without further evidence of recurrence on CT imaging. The patient’s prior obturator lymph node had been noted to remain stable at 6 mm. CT DNA levels at the 1-year mark had recurred to 0.19 MTM/ml but given there was no new evidence of disease, decision was made with the patient to continue active surveillance with close imaging via PET scan and continued CT DNA tracking to assess velocity of rise with consideration of immunotherapy if continued rise.

### CT DNA enhanced surveillance case 2

#### Clinical presentation

Patient B is a 42-year-old female who presented for the evaluation and management of a quickly growing lesion on her right heel. She presented to her dermatologist after noticing recent enlargement but stated that the lesion had been there for more than 5 years. She had noted small changes across the past 2 years but within the past 2–3 months before presentation it began to grow rapidly.

#### Diagnostic workup

Patient underwent resection with dermatology and was found to have acral melanoma, and pathology showed Breslow thickness of 5.7 mm and pathologic staging of T4b. She then underwent WLE and SLE with surgical oncology and was found to have negative margins with no residual melanoma and negative lymph nodes. Finally, she underwent subsequent MRI brain and PET scan which did not show any signs concerning for metastases. Final staging was determined to be stage IIB pT4b N0 M0.

#### Treatment strategy

After discussion with the patient regarding a variety of treatment options including adjuvant immunotherapy with pembrolizumab which is approved for stage IIB melanoma patients, but unfortunately original studies did not include enough acral melanoma patients to decide if this is beneficial and larger pooled clinical trials did not show any benefit to adjuvant PD1 inhibition. Additionally, the benefits of dual agent therapy for metastatic disease with ipilimumab and nivolumab were discussed but this was determined to be too severe of a regimen in the adjuvant setting without metastases.

After discussion with the patient, the decision was made to pursue enhanced surveillance with CT DNA monitoring and recurrent imaging. Plan was made for CT DNA monitoring every 6 weeks with the first 6 months with additional systemic scans via CT as well as an MRI right leg in 3 months. Further discussion for repeat imaging 3–4 times a year during the first year of surveillance.

#### Outcome & follow up

All screening at the 24-month mark including CT DNA monitoring, repeated CT scans as well as repeat PET scan have all returned negative for evidence of recurrent or metastatic disease (with CT DNA levels remaining undetectable). As such at the 24-month mark, decision was made to space out imaging to six-month intervals with continued CT DNA monitoring.

### CT DNA enhanced surveillance case 3

#### Clinical presentation

Patient C is a 48-year-old male who presented for the evaluation and management a longstanding mole on the inside of his right foot. It was located in a partially sun-exposed area of his foot near his sandal line and he noted significant childhood sun exposure. He had no other significant past medical history. Patient noted the lesion along his foot, which had been present for several years, suddenly began to increase in size within the past 3 months.

#### Diagnostic workup

Patient then underwent a biopsy with dermatology and the mass was confirmed to be acral melanoma. Breslow thickness was found to be 3.5 mm and tissue pathologic staging was pT3b. He then went under primary tumor resection, followed by sampling of his groin lymph nodes. Lymph nodes resected were noted to be negative on pathology. Resection showed negative margins and final pathology staging placed him at stage IIC T3b N0 M0. PET scan and brain MRI confirmed there was no concern for metastatic disease.

#### Treatment strategy

In discussion with the patient the possibilities for adjuvant therapy and the lack of evidence in acral melanoma was discussed. Further the role of dual agent immunotherapy in metastatic acral melanoma was discussed as a possibility however the increased risks were additionally elaborated upon. Given his stage IIC status and in discussion with the patient, the decision was made to pursue enhanced surveillance via CT DNA monitoring as well as periodic imaging.

Enhanced monitoring was implemented with systemic and lower extremity imaging in 3 months as well as dermatology surveillance every 3 months and additional CT DNA monitoring every 6 weeks with the first 6 months.

#### Outcome and follow-up

Eighteen months after the initial presentation, CT DNA levels became detectable at 0.5 MTM/ml and imaging showed a right leg subcutaneous node as a likely site of disease recurrence. Management options including initiation of immunotherapy, surgical resection, or SBRT were discussed with the patient. Patient opted to undergo surgical resection with repeat monitoring and consideration of dual agent immunotherapy if surgical clearance was unsuccessful. Surgical resection confirmed acral melanoma and subsequent testing following resection resulted in negative ctDNA post operatively.

Repeat imaging as well as CT DNA testing has remained negative 6 months out from repeat resection. Patient has continued to undergo enhanced surveillance without further evidence of recurrence on imaging or ctDNA monitoring and remains asymptomatic 2 years from initial diagnosis.

### Neoadjuvant management case 1

#### Clinical presentation

Patient D is a 71-year-old male who presented for the evaluation of a lesion on the plantar surface of his right foot that was confirmed by punch biopsy 1 month prior to presentation to be acral lentiginous melanoma at least *in situ*, unable to further assess involvement at deep margin due to issues with biopsy by an outside dermatologist. Patients first noticed the lesion in the plantar forefoot overlying the third metatarsal and stated that lesion has been growing for 1–2 years prior to presentation. It was not painful but occasionally pruritic.

Patient was simultaneously found to have stage I squamous cell carcinoma his vocal cord and was followed with an outside oncologist in vicinity. He underwent IMRT as definitive management for early stage SCC without incident.

#### Diagnostic workup

His ECOG score on presentation was 0 and physical exam was completely within normal limits with the exception of this stated lesion along the plantar surface of the right foot. He underwent a PET scan that showed no evidence of metastatic disease. He underwent an MRI of his right lower extremity and was referred to Orthopedic Oncology after initial presentation for a full wide excision after completion of his vocal cord radiation.

After evaluation by Orthopedic Oncology and MRI of the right foot which showed a progressive superficial soft tissue nodule on the plantar aspect of the forefoot at the level of the second metatarsal head measuring approximately 15 mm in length, 10 mm in width and 6 mm in depth extending adjacent to the flexor tendon of the second digit without discrete involvement. Based on depth observed on MRI, this previously biopsied *in-situ* lesion was felt to more accurately represent a high-risk stage II lesion with at least 6 mm of depth below the skins surface. On discussion at multidisciplinary tumor board consideration of neoadjuvant therapy to preserve the architecture of the forefoot and allow for reduced morbidity of surgery was discussed. As up front surgical management would entail fore foot amputation patient elected for trial of therapy for shrinkage of the tumor site to allow for less aggressive management. Patient underwent neoadjuvant treatment with pembrolizumab q3 weeks with plan for response adapted surgery.

#### Treatment strategy

Patient received three cycles of pembrolizumab in a Q 3-week dosing with the hope of potentially helping spare the foot and reducing morbidity of the surgery needed for negative margins. Repeat MRI showed decreased size and conspicuity of the subtle superficial plantar soft tissue lesion of the forefoot suggestive of treatment response in the patient’s known acral lentiginous melanoma. No major neurovascular, osseous, articular, or tendinous involvement. No new bony or soft tissue mass lesions are identified. Given this patient underwent a right foot melanoma resection with application of a synthetic skin graft substitute and implementation of a wound VAC. Patient tolerated procedure without complication and declined further adjuvant immunotherapy after surgical recovery following a personal life event that required his travel out of the country for an extended period.

He was then transitioned to an active surveillance plan with every 3-month imaging surveillance as well as dermatology follow up and ctDNA monitoring. We implemented simultaneous CT DNA tracking every 6 weeks using surgical samples from resection. Tracking showed an initial value of 0.06 MTM/ml plasma on initial evaluation before trending down to 0 and remaining undetectable.

#### Outcome and follow-up

The patient’s 6-month PET scan showed no evidence of recurrence and treatment plan is to continue CT scans every 6 months for the first 2 years. CT DNA monitoring continued every 6 weeks and remained negative after initial clearance.

### Neoadjuvant management case 2

#### Clinical presentation

Patient E is an 81-year-old female who presented for the evaluation of a dark lesion of the left hallux that was present for several months prior to diagnosis by punch biopsy by an out of hospital podiatrist as PT4bpN0pM0 melanoma. Patient had noticed a recent increase in thickness and size as well as redness overlying the lesions prior to presentation. Her past medical history was relevant for history of breast cancer (s/p lumpectomy and chemo/XRT in 16 years prior), PE on Eliquis, COPD and depression. Patient’s past oncologic history was relevant for a diagnosis 16 years prior to presentation with infiltrating ductal carcinoma 1.1 cm, grade III with extensive ductal carcinoma *in situ*. Breast cancer was ER/PR/HER-2 negative and patient underwent lumpectomy followed by chemotherapy and radiation.

#### Diagnostic workup

Biopsy showed a 4.3-mm Breslow thickness melanoma with positive margins and lymphovascular invasion as well as a Clark Level IV and mitotic index of 3 mm. PET scan showed no sites of distant metastases, and the patient was referred for further evaluation and treatment.

#### Treatment strategy

Patient was initially hesitant to proceed directly with up front amputation and wanted to attempt toe/sparing interventions. She was discussed at multi-disciplinary tumor board and determination for attempt at neoadjuvant immunotherapy to shrink tumor and minimize aggressive surgical management was recommended and discussed with patient. Patient ultimately opted for neoadjuvant therapy and underwent four cycles of pembrolizumab and continuous CT DNA testing monitoring which returned negative. Patient experienced some evolution in the distal toe at the biopsy site with treatment but based on persistent disease following four cycles of immunotherapy she underwent surgical resection. Surgical resection and sentinel lymph node sampling was performed and showed a pT4b lesion and one lymph node was removed and positive, final stage IIIC. Based on positive node and lack of tumor reduction discussion was made to increase immunotherapy from single agent pembrolizumab but based on comorbidities and patient developing subsequent post operative surgical wound infection, she was maintained on 1 year of adjuvant immunotherapy with pembrolizumab. Repeat PET scan showed no evidence of further recurrent or active melanoma.

#### Outcome and follow-up

Interestingly patients’ ctDNA level remained negative throughout neoadjuvant and adjuvant period even with nodal positive disease and residual tumor at the primary site. Patient since completed 1 year of adjuvant immunotherapy with negative ctDNA monitoring through treatment and negative imaging q3 months, 1 year out from time of surgical resection with no evidence of disease.

## Discussion

### Prognostic implications

The prognostic impact of adjuvant and neoadjuvant immunotherapy in acral melanoma is still being elucidated. As seen above there are few studies that include specific data for acral melanoma patients and as a result insufficient powered study to draw specific conclusions. Many treatment guidelines are based around studies in cutaneous melanoma patients. Some studies suggest that achieving a pCR with neoadjuvant therapy is associated with improved survival outcomes. Sharon et al. showed that in patients treated with pembrolizumab in stage III/IV cutaneous melanoma showed a 95.4% survival if they achieved pCR versus 57% for those with a nonresponse (22). However, others indicate that the benefits of immunotherapy may be limited to specific subgroups of patients based on molecular and genetic profiles (23). Silva et al. showed melanomas with BRAF V600E and V600K mutations, those with V600K had a higher mutational load and responded better to anti PD-1 therapy (23). These studies unfortunately do not specify results in the acral melanoma population and as such further study is necessary to verify these results in this sub-population.

When comparing the effectiveness of neoadjuvant versus adjuvant immunotherapy, it is crucial to consider the possibility of lead time bias. Lead time bias occurs when early detection or treatment of a disease appears to improve survival times without actually affecting the overall course of the disease. In the context of neoadjuvant therapy, patients receive treatment before surgery, potentially leading to earlier detection of treatment response and survival metrics that appear more favorable compared to adjuvant therapy, which is administered post-surgery. This earlier intervention can create the illusion of prolonged survival merely because the disease is being addressed sooner, not necessarily because the therapy is more effective. Studies need to account for this bias by using appropriate statistical methods and study designs that compare survival from a common starting point, such as the time of diagnosis or surgical intervention, rather than the initiation of therapy. Understanding and adjusting for lead time bias is essential to accurately assess the true benefits of neoadjuvant versus adjuvant chemotherapy in acral melanoma. Our own small sample size echoes the potential benefits for neoadjuvant therapy in setting of tumor reduction and reducing surgical morbidity to treatment but also shows the shortcomings of single agent PD-1 therapy for all acral melanoma patients.

### Comparative effectiveness of adjuvant versus neoadjuvant chemotherapy

Comparative studies between adjuvant and neoadjuvant therapies in acral melanoma are limited, but available evidence suggests that neoadjuvant therapy may offer certain advantages. Trials with data regarding acral melanoma are rare but the SWOG trial demonstrated neoadjuvant therapy plus adjuvant therapy with pembrolizumab improved event free survival compared to adjuvant alone but did not have sufficient patients to draw conclusions regarding acral melanoma specifically.

Adjuvant studies additionally have shown mixed results with Li et al. showing no benefit even compared to interferon therapy in acral melanoma while Zhong et al. saw benefit with better relapse control with ATP kinase inhibitors dabrafenib & trametinib compared to PD-1 immunotherapy but specifically in BRAF-mutant melanomas. Arak et al. compared different variants of adjuvant therapy but suggested no statistically significant difference among them however a failure to reach sufficient DFS events within the monitoring period suggests PD-1 inhibitors may have favorable outcomes compared to other systemic therapies but this effect could not be quantified within the monitoring period of the study. Mao et al. compared variants of interferon therapy dosing have since fallen out of practice in favor of immunotherapy. This has occurred as a result of PD-1 inhibitor’s superior efficacy in the treatment of melanoma in general as well as lower constitutional and psychiatric toxicity (interferon therapy is associated with severe fatigue, fevers, myalgias, depression, hepatotoxicity and myelosuppression); however, as we see from the above trials, acral melanoma continues to be an area of needed research (24).

Neoadjuvant therapy can potentially downstage the tumor, making surgical resection more feasible and less extensive (15,254. For example, Amaria et al. (2022) found that neoadjuvant therapy with relatimab and nivolumab resulted in a 57% pCR and an overall 70% pathologic response rate facilitating significant tumor downstaging in higher degree stage III resectable or stage IV cutaneous melanomas (25). Furthermore, neoadjuvant treatment allows for the assessment of treatment response, which can provide prognostic information and guide postoperative therapy (20). However, the direct comparison of adjuvant and neoadjuvant chemotherapy specifically for acral melanoma requires further research.

Both adjuvant and neoadjuvant therapies carry risks of adverse effects. Immunotherapy-related toxicities, including myelosuppression, neuropathy, and gastrointestinal symptoms, can impact patient quality of life and treatment adherence. The timing of chemotherapy (adjuvant versus neoadjuvant) may influence the overall burden of these side effects and the patient’s ability to tolerate subsequent treatments (26). A systematic review and meta-analysis by Fujiwara et al. showed that neoadjuvant immune check point inhibition was not associated with significant increase in incidence of treatment related death or grade 3–4 adverse events while immune check point inhibition in the adjuvant setting was (27).

### Role of circulating tumor DNA testing

Circulating tumor DNA (ctDNA) testing is an emerging non-invasive method that detects fragments of DNA shed by tumors into the bloodstream with higher tumor burden and the presence visceral metastases increasing ctDNA levels (28). This technology offers several potential benefits for the monitoring and management of acral melanoma patients. ctDNA testing can be used to monitor disease progression and response to therapy. Studies have shown that ctDNA levels correlate with tumor burden and can provide early indications of treatment efficacy. For example, a study by Lee et al. (2017) demonstrated that ctDNA levels decreased in response to effective therapy and increased with disease progression in general melanoma patients (28). This real-time monitoring capability allows for more timely adjustments in various treatment strategies.

Detecting minimal residual disease (MRD) after surgical resection is crucial for identifying patients at high risk of recurrence. ctDNA testing can detect MRD with high sensitivity, providing a valuable tool for post-surgical surveillance. A study by Tan et al. (2019) found that ctDNA was a significant predictor of recurrence in general melanoma patients, with detectable ctDNA indicating a higher likelihood of relapse (309). In our surveillance cases, this monitoring added an extra layer of screening past the gold standard that led to less delay in imaging and subsequent intervention.

ctDNA testing can also guide the selection and timing of adjuvant and neoadjuvant therapies. For instance, the presence of ctDNA post-surgery may indicate the need for more aggressive adjuvant therapy. Conversely, a significant reduction in ctDNA levels during neoadjuvant therapy could suggest effective tumor response and inform decisions about surgical intervention timing. In our patients treated with neoadjuvant immunotherapy the downstaging of the tumor in conjunction with discussions with surgical teams and the patients allowed for less invasive surgeries and even limb sparing. As with any therapy, no test is perfect and not all disease results in positive values. In the setting of patient E, there was no detectable ctDNA at any point during therapy even through path sampling confirming both residual disease at the primary site and microscopic nodal positivity.

The molecular profiling capabilities of ctDNA allow for the identification of specific genetic mutations and alterations, which can guide personalized treatment plans. A study by Abbosh et al. (2017) highlighted the prognostic value of ctDNA in melanoma, demonstrating that patients with detectable ctDNA had worse overall survival compared to those without detectable ctDNA (29). Unfortunately, as the literature currently stands, the data discussed above has been generalized from general melanoma and further study will be needed to verify these findings in patients with acral melanoma. This information can help clinicians tailor treatment plans to individual patient profiles, potentially improving outcomes by potentially assisting with decisions on timing of therapy as well as intensity.

Despite the discussed potential ctDNA in its current state has several technical limitations. Exceedingly small amounts of ctDNA within a background of cell free DNA pose a challenge and necessitates the use of highly sensitive methods of detecting mutations which in turn can impact sensitivity and specificity since they are limited by the error rates of DNA polymerase sequencing (30). They additionally vary with different assays but typically have reliable detection above a variable allele frequency of 0.5% (31). Further detection can become unreliable if input material is limited and lead to false negatives, especially in early stage disease (31). The presence of multiple tumor clones with distinct genetic profiles creates another challenge that requires the use of comprehensive ctDNA assays to capture all the tumor-derived mutations (32). Additionally, the potential for contamination with normal DNA due to lysis of cells creates a need for rapid processing within 1h–4h after samples are drawn, unless specialized collection tubes are used which in turn adds an additional cost (33). Of note, FoundationOne Liquid CDx Assay has achieved FDA approval.

## Conclusion

Acral melanoma presents unique challenges in diagnosis and treatment, necessitating specialized therapeutic strategies. While surgical excision remains the cornerstone of treatment, the roles of adjuvant and neoadjuvant therapies are evolving. Immune checkpoint inhibitors and targeted therapies are increasingly favored in the adjuvant setting due to their efficacy and survival benefits. Neoadjuvant therapy shows promise in downstaging tumors and providing prognostic insights, though its role in acral melanoma requires further validation through clinical trials.

ctDNA testing is a promising tool for monitoring disease progression, detecting MRD, and guiding personalized treatment plans. This could be implemented as an add-on in the form of enhanced surveillance to the existing gold standard therapy, especially in early stage melanomas where active surveillance is the standard of care. This has a likely increased potential benefit in acral melanoma patients where the tumors tend to be more aggressive; however, given the lack of early recurrence in our patients with this method, it served primarily as additional peace of mind for patients and served as an additional method of detection. Many of these advantages lend credence to the potential of personalized treatment plans although continued follow-up and larger scale review are necessary.

Comparative studies focusing on the efficacy, safety, and long-term outcomes of adjuvant versus neoadjuvant immunotherapy will be essential to optimize treatment protocols for this rare and aggressive melanoma subtype. As it stands now there is a dearth of literature regarding both adjuvant and neoadjuvant immunotherapy in this patient population and will be a focus of future studies. Given the lack of clear guidelines for these patients in early stage acral melanoma future research will need to be focused on evaluation of combination therapies, new biomarkers aside from ctDNA as well as optimizing treatment doses and regimens with existing check point inhibitors.

## Data Availability

The original contributions presented in the study are included in the article/supplementary material. Further inquiries can be directed to the corresponding author.
